# Tracing the Flight: Investigating the Introduction of Avian Metapneumovirus (aMPV) A and B

**DOI:** 10.3390/ani14121786

**Published:** 2024-06-14

**Authors:** Giovanni Franzo, Matteo Legnardi, Giulia Faustini, Riccardo Baston, Francesca Poletto, Mattia Cecchinato, Claudia Maria Tucciarone

**Affiliations:** Department of Animal Medicine, Production and Health, University of Padova, 35020 Legnaro, Italy; matteo.legnardi@unipd.it (M.L.); giulia.faustini.1@phd.unipd.it (G.F.); riccardo.baston@phd.unipd.it (R.B.); francesca.poletto.1@studenti.unipd.it (F.P.); mattia.cecchinato@unipd.it (M.C.); claudiamaria.tucciarone@unipd.it (C.M.T.)

**Keywords:** aMPV, molecular epidemiology, phylogeography, North America, wild birds

## Abstract

**Simple Summary:**

Avian metapneumovirus (aMPV) causes substantial economic losses globally. Different aMPV subtypes circulate in various regions, with subtypes A and B prevalent in the Old World and aMPV-C in North America. Recently, aMPV-A and aMPV-B have been detected in the U.S., raising questions about their introduction pathways. This study used phylodynamic and phylogeographic analyses of the G protein sequences to investigate potential importation routes. Findings suggest that aMPV-B in the U.S. likely originated from Eastern Asian strains related to European ones, with wild bird migration through the Beringian crucible being a probable pathway, similarly to avian influenza. aMPV-A appears to have Mexican origins, with strains related to Asian ones, pointing again to wild bird migration rather than trade or illegal importation. Given the limited information on wild birds’ role in aMPV spread and the significant impact on the poultry industry, further wild bird surveys are recommended.

**Abstract:**

Avian metapneumovirus (aMPV) has been identified as an important cause of respiratory and reproductive disease, leading to significant productive losses worldwide. Different subtypes have been found to circulate in different regions, with aMPV-A and B posing a significant burden especially in the Old World, and aMPV-C in North America, albeit with limited exceptions of marginal economic relevance. Recently, both aMPV-A and aMPV-B have been reported in the U.S.; however, the route of introduction has not been investigated. In the present study, the potential importation pathways have been studied through phylogenetic and phylodynamic analyses based on a broad collection of partial attachment (G) protein sequences collected worldwide. aMPV-B circulating in the U.S. seems the descendant of Eastern Asian strains, which, in turn, are related to European ones. A likely introduction pathway mediated by wild bird migration through the Beringian crucible, where the East Asian and Pacific American flight paths intersect, appears likely and was previously reported for avian influenza. aMPV-A, on the other hand, showed a Mexican origin, involving strains related to Asian ones. Given the low likelihood of trade or illegal importation, the role of wild birds appears probable also in this case, since the region is covered by different flight paths directed in a North–South direction through America. Since the information on the role of wild birds in aMPV epidemiology is still scarce and scattered, considering the significant practical implications for the poultry industry demonstrated by recent U.S. outbreaks, further surveys on wild birds are encouraged.

## 1. Introduction

Avian metapneumovirus (aMPV) is a highly infective virus that affects turkeys and chickens, causing turkey rhinotracheitis (TRT) and swollen head syndrome (SHS), respectively. aMPV also has the capacity to infect other avian species [1,2,3]. The virus is primarily associated with upper respiratory infections, predisposing birds to opportunistic bacterial pathogens, which can result in severe respiratory disease, high morbidity, and mortality rates. Furthermore, aMPV can infect the reproductive system, leading to lower egg production and decrease in quality [4].

aMPV is identified as a single-stranded, non-segmented, enveloped, and negative-sense RNA virus, with a size range of approximately 13.3–14 kb, belonging to the family *Pneumoviridae* and genus *Metapneumovirus*. Its genome comprises eight genes arranged in the 5′-3′ direction as follows: Nucleoprotein (N), Phosphoprotein (P), Matrix (M), Fusion (F), Matrix 2 (M2), Small Hydrophobic (SH), Attachment (G), and Large Polymerase (L) [5].

The attachment (G) and fusion (F) proteins are major structural components located on the virus envelope and play crucial roles in the virus interactions with the host cell [6,7]. The G protein, in particular, is vital for virus attachment to the cell membrane and contains major neutralizing epitopes. Its biological role, along with the viral high evolutionary rate, contributes to the genetic heterogeneity of this region. As a result, the G protein region is often targeted for genotypic characterization, phylogenetic, and molecular epidemiological studies, making it the most frequently sequenced region of the aMPV genome [8].

aMPV has long been acknowledged as responsible for production drop and economic losses within the global poultry industry, but its significance seems to have grown in recent years. aMPV subtype A was first identified in South Africa in the 1970s and subsequently spread to several European countries [9,10]. Around the same time, another subtype, aMPV-B, was recognized and gradually became dominant in the Old World [1,4,8]. The U.S. was unaffected until 1996, when aMPV subtype C emerged in turkeys and wild birds [11,12,13]. aMPV-C is predominantly established in North America and was thereafter sporadically detected in Europe and Asia [3,12,14,15,16,17]. However, North American and Eurasian AMPV-C strains belong to two separate lineages, with the former showing tropism primarily for *Galliformes* and the latter for anatids [18]. Another subtype, aMPV-D, was identified only in retrospective French samples from the 1980s [19]. Although two new subtypes were detected in North America in black-backed gulls and monk parakeet chicks [20,21], aMPV-C remained the principal subtype affecting U.S. flocks until very recently, when aMPV-B spread to the U.S. [22]. Additionally, sequences of aMPV-A from strains collected in the U.S. have recently been published (GenBank Acc. Numbers: PP442011 and PP442012). The introduction of these two subtypes in the US represented a challenge for their identification, highlighting a potential weakness in the surveillance/diagnostic system. Moreover, the circulation in a naïve population led to extremely severe clinical signs, whose control has not been promptly effective, resulting in astonishing economic losses. While focusing on the significant clinical and productive implications, the sources of aMPV introduction into North America have not been investigated yet. The generally limited and scattered sampling and sequencing efforts impede the establishment of direct epidemiological connections on a global scale. However, some techniques based on molecular epidemiology and biostatistics allow us to partially compensate for this lack of information. To this purpose, the present study employs phylogenetic and phylogeographic approaches to estimate the source and the timing of introduction of aMPV subtypes A and B into the U.S.

## 2. Materials and Methods

### 2.1. Dataset Preparation

All aMPV-A and -B sequences of the partial G gene for which the date and country of collection were known were downloaded from GenBank. After a quality check (i.e., poor alignment, unknown bases, premature stop-codons or frameshift mutations suggestive of sequencing errors), only suitable sequences were selected for the analysis. Independent datasets were generated for the two aMPV subtypes. The analyzed region was selected to achieve the best compromise between sequence length and country-year representativeness. After sequence alignment with MAFFTv7 [23], a tree was reconstructed using IQ-Tree v2.3.4 [24]. The selection of the substitution model was based on the Bayesian Information Criteria (BIC) calculation, using the same software. All strains clustering with vaccine ones were identified and removed from the study.

### 2.2. Phylodynamic and Phylogeographic Analysis

The Bayesian serial coalescent approach implemented in BEAST 1.10 was used to reconstruct several population parameters, including time to the most recent common ancestor (tMRCA), evolutionary rate, and viral population dynamics [25]. For each dataset, the nucleotide substitution model was selected based on the BIC score calculated using JmodelTest [26]. The molecular clock was selected, calculating the marginal likelihood estimation through path-sampling and stepping-stone methods, as suggested by Baele et al. [27]. The non-parametric Bayesian Skygrid [28] was selected to reconstruct viral population changes over time (relative genetic diversity: effective population size∙generation time; N_e_∙τ). A discrete state phylogeographic analysis was also performed as described by Lemey et al. [29], implementing an asymmetric migration model with Bayesian stochastic search variable selection (BSSVS), allowing the identification of the most parsimonious description of the spreading process and calculating a Bayesian Factor (BF) indicative of the statistical significance of the inferred migration path between geographic areas. The BF for a particular rate k contributing to the migration graph is calculated as the posterior odds that rate k is non-zero divided by the equivalent prior odds. The default truncated Poisson prior with mean µ = ~log 2, which assigns 50% prior probability on the minimal rate configuration (k-1), was selected. Due to the sparse nature of the sequence-country combination and the likely missing sampling in several countries, and in order to obtain a more balanced dataset, the analysis was also performed aggregating countries in macro-areas based on their spatial proximity and considering geopolitical factors (i.e., Africa, Asia, Central America, Europe, Middle East, North America, and South America). Two independent runs of 200 million generations were performed and the log and tree files were merged using logcombiner after the removal of a burn-in of 20%. Results were analyzed using Tracer 1.7 and runs were accepted only if the estimated sample size (ESS) of the different parameters was greater than 200. Convergence and mixing were also visually inspected. Parameter estimation was summarized in terms of mean and 95% highest posterior density (HPD). Maximum clade credibility (MCC) trees were constructed and annotated using TreeAnnotator (BEAST package). SpreaD3 [30] was used to calculate the BF associated with each migration route. A BF greater than 10 was selected as a statistically significant threshold for transition rates among countries. Additional summary statistics and graphical outputs were generated using homemade R scripts [31].

## 3. Results

A total of 45 and 538 partial G sequences were included in the aMPV-A and aMPV-B dataset.

The aMPV-A tMRCA ancestor was estimated in 1984 [95HPD: 1956.25–1988.00], with an evolutionary rate of 2.83 × 10^−3^ [95HPD: 7.72 × 10^−4^–5.48 × 10^−3^].

**Figure 1:**
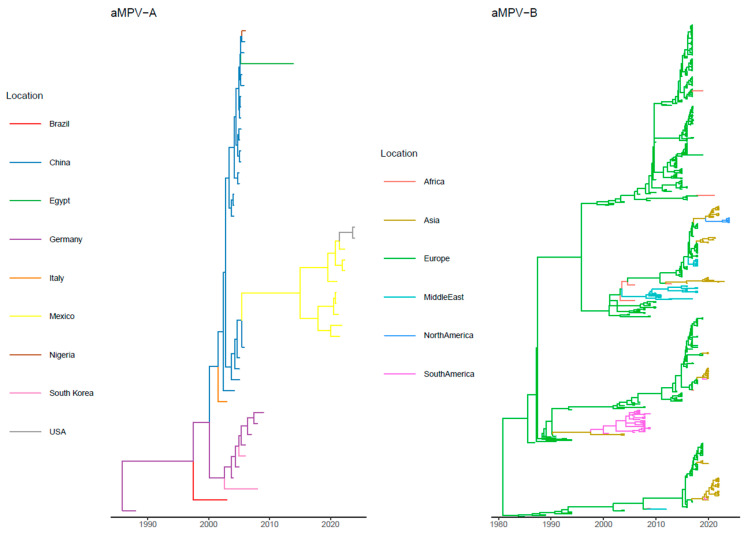
Maximum clade credibility tree estimated based on the partial G-gene alignment of aMPV-A (**left**) and aMPV-B (**right**). Branches have been color-coded based on the strain sampling regions and predicted ancestral locations. Further details, including strain name and location posterior probability, are available in Supplementary Figures S1 and S2.

The introduction in Mexico, apparently from Asian countries (i.e., China) (Figure 1 and Supplementary Figure S1), occurred in the first years of the new century (approximately 2005), while the following spread to North America was estimated around 2020. Well-supported migration rates connected Europe to the Asian region, Asia to Africa and South and North America. In more detail, a well-supported migration rate was inferred between Mexico and USA.

**Figure 2:**
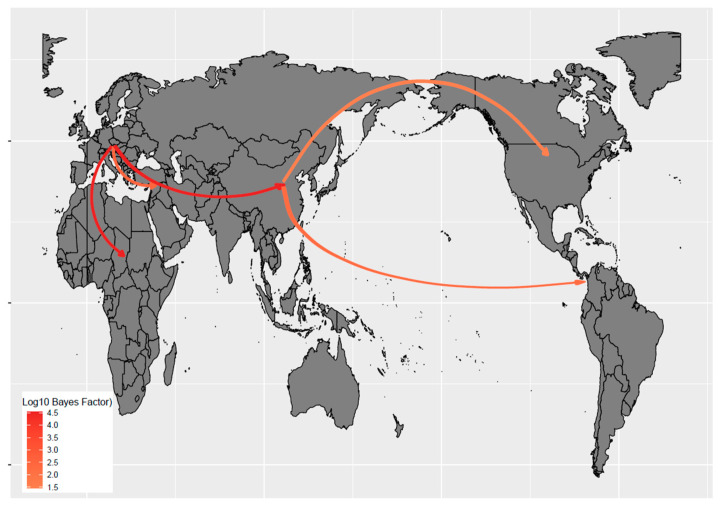
aMPV-B genotype migration paths. Well supported migration paths (i.e., BF > 10) among areas are depicted. The arrows indicate the directionality of the process, while the edge color is proportional to the base-10 logarithm of the Bayesian factor. The location of each area has been matched with its centroid.

The tMRCA of aMPV-B was inferred in 1979.76 [95HPD: 1935.06–1984.99], and the evolutionary rate was comparable to the aMPV-A one (i.e., 3.52 × 10^−3^ [95HPD: 2.17 × 10^−3^–9.94 × 10^−3^]). The phylogeographic analysis of aMPV-B estimated that the North American strains likely originated from Eastern Asia. Based on MCC tree investigation, Thailand was the most likely source. Statistically supported migration rates were proven between Asia and both North and South America (Figure 2). The tMRCA of the U.S. clade was predicted around 2022 [95HPD: 2021.6–2023.7] (Figure 1 and Supplementary Figure S2).

## 4. Discussion

While the distribution of aMPV subtypes remained stable for decades, the recent report of subtype aMPV-A and aMPV-B spreading in North America raised considerable concern for the local poultry sector, posing new practical and theoretical questions on infection introduction and containment.

The phylogeographic analysis of aMPV-B estimated that the North American strains likely originated from Eastern Asia, particularly Thailand. The time to the most recent common ancestor (tMRCA) of the U.S. clade was predicted around 2022 (Figure 1 and Supplementary Figure S2), which testifies to the relatively swift identification of the viral emergence. The most closely related strains from Thailand were sampled in 2021, and the tMRCA of the overall clade was estimated in 2019 (Supplementary Figure S2). The relatively long time elapsed—at least from an RNA virus evolution perspective—between the detection of these two groups weakens the strength of inference regarding the introduction route. However, avian influenza virus (AIV) migration from Eastern Asia to North America has been previously reported multiple times and has been mediated by migratory birds with overlapping flight paths [32,33,34]. The East Asian and Pacific American flight paths intersect in the regions of Eastern Russia and Alaska around the Bering Strait. It is estimated that about 1.5–3 million aquatic birds move from Asia to Alaska annually during the breeding season, providing a significant potential for viral dispersal [35,36]. Interestingly, connections between Eastern Europe and Asia, reportedly mediated by wild birds for AIV [32], have also been inferred in this study for aMPV-B, supporting the relevance of this spreading mechanism (Figure 2).

aMPV detections in wild birds, although limited and sporadic, have been reported for several aMPV subtypes, including aMPV-B [2,3,11,21,37]. Moreover, increasing evidence suggests that some wild bird taxa could play a role in aMPV epidemiology. Particularly, wild ducks, geese, gulls, and pheasants have proven to be susceptible to aMPV [2]. Due to their host ecology, these birds might act as viral carriers or reservoirs.

Wild birds were already demonstrated to play a role in aMPV subtype C maintenance in the U.S., where the infection was serologically confirmed in American coots, American crows, Canada geese, cattle egrets, and rock pigeons [11]. aMPV-C was detected in geese, sparrows, and starlings sampled in areas neighboring turkey farms, confirming that wild birds could act as sources of infection for domestic turkeys [38]. Waterfowl has also been found positive in Canada [39], widening the areas where wild birds can cross paths over different flyways.

Although other pathways cannot be excluded, their likelihood seems lower. With the U.S. primarily being an exporter, the role of live animal importation should be discounted, and the illegal importation of poultry or poultry products into North America seems unlikely due to the difficulty of smuggling live birds or uncooked products with current transportation biosecurity measures [32]. The involvement of the Atlantic Rim in virus importation from Europe to North America through wild bird migration has recently been proposed for AIV [40]. However, the occurrence of such events seems lower, and this hypothesis is further questioned by the mediation of Asian countries herein reported.

Following its introduction in North America, other factors could have been involved in the spreading process. This region is covered by different North-to-South flight paths through America (i.e., the Pacific, Central, Mississippi, and Atlantic flyways). The within-flyways AIV transmission is well established, and inter-flyways viral passages can occur, albeit at a lower rate. Interestingly, the Alaskan region is in connection with localities belonging to all American flyways, confirming its relevance as a hub for wild bird (and associated virus) migration [41].

Such flyways might also have been involved in the introduction of aMPV-A from Mexico to the U.S., as inferred using the performed phylogeographic analysis (Figure 1 and Supplementary Figure S1). Mexico is also on the route of all American flyways, which converge and overlap on this country [42]. Unfortunately, the limited number of sequences and the long branch separating the Mexican–U.S. clade from the remaining strains prevent reliable conclusions. The direct introduction of aMPV from Asia to Mexico through wild bird migration can be excluded. Potentially, aMPV-A might have followed the same pathway as aMPV-B, being introduced from the Beringian crucible and then migrating southward without establishing in the U.S. After successfully infecting the Mexican poultry population, it might have spread northwards during subsequent migratory seasons. However, the contribution of the legal or illegal poultry trade or importation cannot be excluded with confidence also in this case. Moreover, aMPV-A and B detections in wild birds from South America were reported [43,44]. Of note, a statistically significant migration route was inferred from Asia (South Korea) to South America (Brazil), involving a lineage of European origin (Figure 1 and Supplementary Figure S2).

Since the information on the role of wild birds in aMPV epidemiology is still scarce and scattered and considering the significant practical implications for the poultry industry demonstrated by recent U.S. outbreaks, further surveys of wild birds are encouraged. A better understanding of the poultry/wild bird interface in aMPV epidemiology and a more comprehensive characterization of its host range extent are needed.

## 5. Conclusions

The present study reconstructs the introduction route and timing of the aMPV-A and B in North America. In both instances, Asian countries were predicted as the most likely sources. However, because of the absence of live animal importations from these regions, other sources should be considered, with wild birds being the most likely suspected. However, the data on the role of wild birds in aMPV epidemiology are still scarce and sparse. Therefore, this study emphasizes the need for continued surveillance and a better understanding of the role of wild birds in aMPV epidemiology. Given the significant impact on the poultry industry, further research is recommended to explore the poultry/wild bird interface and to comprehensively characterize the host range of aMPV. This would help the development of more effective strategies for managing and containing the virus, mitigating its impact on both wild and domestic bird populations.

## Data Availability

All data were retrieved from the freely available GenBank dataset, and the relative accession numbers are provided in the Supplementary Material.

## Supplementary Materials

The following supporting information can be downloaded at: https://www.mdpi.com/article/10.3390/ani14121786/s1, Supplementary Figure S1. Maximum clade credibility tree estimated based on the partial G-gene alignment of aMPV-A. Branches have been color-coded based on the strain sampling regions and predicted ancestral locations, whose posterior probability is reported nearby the corresponding node. Supplementary Figure S2. Maximum clade credibility tree estimated based on the partial G-gene alignment of aMPV-B. Branches have been color-coded based on the strain sampling regions and predicted ancestral locations, whose posterior probability is reported nearby the corresponding node.
