# Supplementary figures and images for: Tracing the Flight: Investigating the Introduction of Avian Metapneumovirus (aMPV) A and B
Source: Animals (Basel). 2024 Jun 14;14(12):1786. doi: 10.3390/ani14121786 (PMC11200574; doi:10.3390/ani14121786)

## Location

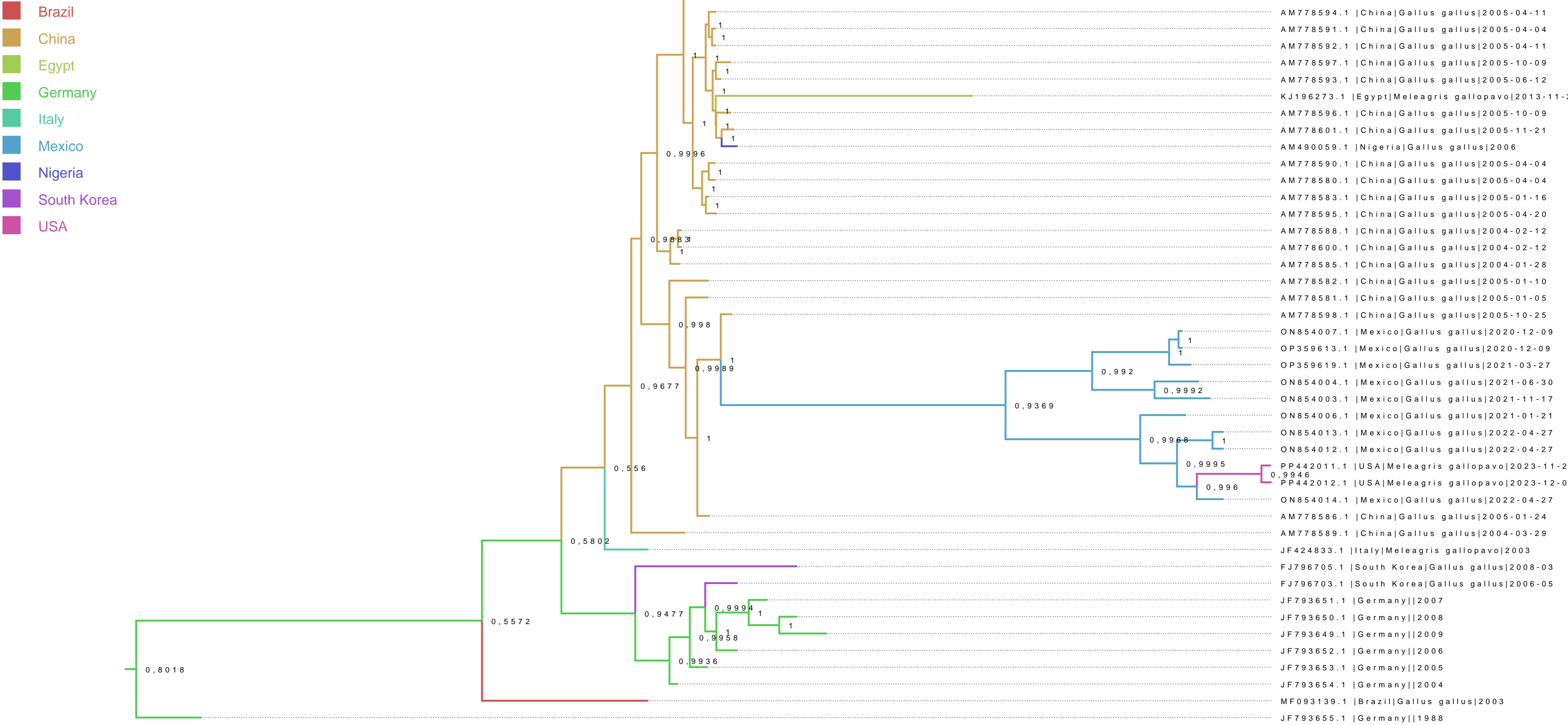

|    |      |      |      |      |      |
|----|------|------|------|------|------|
| 80 | 1990 | 2000 | 2010 | 2020 | 2030 |
|----|------|------|------|------|------|

Supplement: Supplementary file 1 [file animals-14-01786-s001.zip › Supplementary figure S1.pdf]

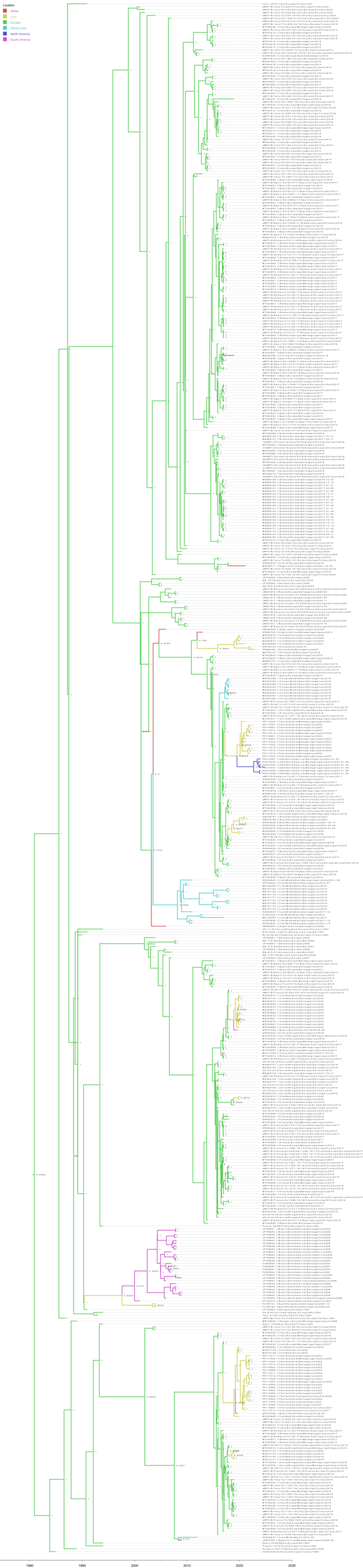

Supplement: Supplementary file 1 [file animals-14-01786-s001.zip › Supplementary figure S2.pdf]
